# Orientation Effects in Ballistic High-Strained P-type Si Nanowire FETs

**DOI:** 10.3390/s90402746

**Published:** 2009-04-17

**Authors:** Jia-Hong Zhang, Qing-An Huang, Hong Yu, Shuang-Ying Lei

**Affiliations:** 1 Key Laboratory of MEMS of Ministry of Education, Southeast University, 2 Sipailou, Nanjing, Jiangsu, People’s Republic of China; E-mails: h_yu@seu.edu.cn; lsy@seu.edu.cn; 2 College of Electronic & Information Engineering, Nanjing University of Information Science & Technology, 219 Ning-Lu Road, Nanjing, Jiangsu, People’s Republic of China; E-mail: zjhseu_nj@yahoo.com.cn;

**Keywords:** NW FET pressure sensor, orientation, uniaxial stress, ballistic hole transport

## Abstract

In order to design and optimize high-sensitivity silicon nanowire-field-effect transistor (SiNW FET) pressure sensors, this paper investigates the effects of channel orientations and the uniaxial stress on the ballistic hole transport properties of a strongly quantized SiNW FET placed near the high stress regions of the pressure sensors. A discrete stress-dependent six-band k.p method is used for subband structure calculation, coupled to a two-dimensional Poisson solver for electrostatics. A semi-classical ballistic FET model is then used to evaluate the ballistic current-voltage characteristics of SiNW FETs with and without strain. Our results presented here indicate that [110] is the optimum orientation for the p-type SiNW FETs and sensors. For the ultra-scaled 2.2 nm square SiNW, due to the limit of strong quantum confinement, the effect of the uniaxial stress on the magnitude of ballistic drive current is too small to be considered, except for the [100] orientation. However, for larger 5 nm square SiNW transistors with various transport orientations, the uniaxial tensile stress obviously alters the ballistic performance, while the uniaxial compressive stress slightly changes the ballistic hole current. Furthermore, the competition of injection velocity and carrier density related to the effective hole masses is found to play a critical role in determining the performance of the nanotransistors.

## 1. Introduction

Based on CMOS-MEMS technology [1], micro FET sensors such as the CMOS humidity sensor [2,3] and the CMOS pressure sensor [4,5], are an interesting topic of investigation in terms of the development of miniaturized and high performance sensors. As device sizes further shrink towards the nanoscale, existing CMOS devices will evolve from planar to three-dimensional non-planar NW devices. Recently, SiNWs have attracted considerable attention among the device and circuit engineering community due to their potential applications as nanoscale MOSFETs and nanosensors for further nanoelectronic and nanoelectromechanical system [6–9], which will operate under strong quantum confinement and high-strain regimes. Therefore, in order to fully assess the ultimate performance of SiNW FETs, as well as the physics of nanosensors, proper modeling is essential in understanding the electrical characteristics of the high-strained SiNW transistor. A first important step is the calculation of the transport properties of SiNWs. For instance, Wang *et al*. [10] have theoretically investigated the effects of the band structure, NW diameter and carrier type on the transport performance limits of unstrained ballistic SiNWs transistors with the transport axis along [100], but SiNWs with various transport orientations (e.g., [100], [110], [111], [112]) have been synthesized by different experimental groups [6,11,12]. In particular, SiNW transistors of diameters even down to 3 nm have already been demonstrated [13]. Because of the device’s very small dimensions, the atoms in the cross section of SiNWs will be countable, and bond orientation, crystal symmetry, and quantum confinement will matter. Thus, understanding the effect of wire orientations and quantum confinement on the ballistic transport in SiNWs is becoming increasingly important [14,15]. Meanwhile, strain engineering takes a key position among other technological innovations since the beneficial effect of strain on device performance is comparatively large [16]. In order to analyze the sensitivity of FET pressure sensor, the relationship between the drive current and the stress needs to be examined.

In this paper, based on the theory developed by Luttinger and Kohn [17], the bandstructures of p-type silicon nanowires are calculated by using a two-dimensional (2-D) discrete six-band k.p method [18–22] that quantitatively takes into account hole quantization effect, band mixing effect, spin-orbit coupling effect as well as mechanical strain. Then we apply a top-of-the-barrier ballistic FET model [23] to explore the effects of the orientation and the stress on ballistic hole transport properties of the p-type SiNW FETs. We find that the SiNW FET performance displays a strong orientation dependence, however, the effect of strain on the ballistic drain current of the ultra-scaled cross section SiNW FETs with the [110] and [111] orientations, except for the [100] orientation, turns out negligible, which can be attributed to an only slight variation of the hole effective mass due to the strong quantum confinement. However, for larger NW transistors with various transport orientations, the uniaxial tensile stress obviously alters the ballistic performance. Furthermore, the competition of injection velocity and carrier density related to the effective hole masses is found to play a critical role in determining the performance of the nanotransistors.

## 2. Theory Model

**Figure 1.**
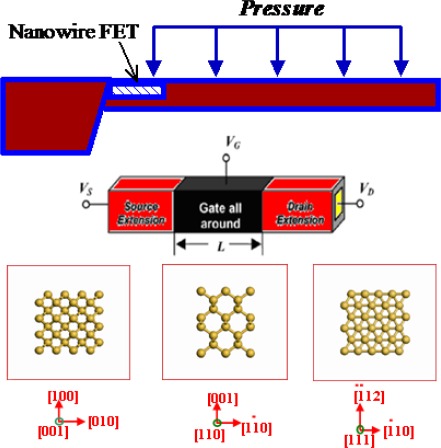
A schematic diagram of the SiNW FET pressure sensor. In this work, the SiNW FETs with various channel orientations i.e., [100], [110], [111] are explored.

As shown in Figure 1, the device that we have studied is a p-type gate-all-around SiNW MOSFET separated by the silicon dioxide layer from the metallic gate (to achieve the same OFF-current for different devices, the work function of the gate material is adjusted), which is the most important component of the SiNW FET pressure sensor. To obtain the maximum sensitivity, the SiNW FET should be placed near the anchor of the FET pressure sensor, which is the high stress region. To investigate how the stress and the orientation affect the ultimate device performance of SiNWs FET, in this work, the strained square SiNW FETs (transport along the *z* direction) with various channel orientations i.e., [100], [110], [111] are explored, and the Si body thickness, T_Si_, is assumed to be equal to the wire width, W_Si_.

In order to investigate the ballistic hole transport properties of SiNW FETs, we have to calculate the valence subband *E_n_* (*k_z_*) and wave function *φ*_*n*,*k*_*z*__ (*x*, *y*) of the SiNW. It is well-known that the k.p theory is a well established method to describe the bandstructure. Despite its field of application should be restricted to not very small systems, the amazing correspondence between experimental and numerical results obtained by the k.p method suggests that the limits of validity of the method are beyond what it might be expected, reaching good descriptions of nanoscale systems [24–26]. In this work, using material parameters as listed in Table 1, the subband structures and wave functions of the unstrained and stained silicon are first calculated by using the stress-dependent six-band k.p model under the triangular-well approximation, which is a powerful tool for quantitative evaluation of strain-induced effective mass changes.

The Luttinger parameters we use well reproduce the bulk *E-k* relations for silicon. For the SiNW, whose channel orientation is along the *z* direction, holes are confined in the *x* and *y* directions, which requires a discrete treatment. In other words, at a grid point along the quantization directions in the real space, the stress-dependent six-band k.p Schrödinger equations need to be discretized with the nine-point finite difference method:

(1) $$[H_{\mathrm{kp}}(k_{x}=-i\partial /\partial x,k_{y}=-i\partial /\partial y,k_{z})+H_{\mathrm{strain}}+H_{\mathrm{so}}+V(x,y)]\varphi_{n,k_{z}}(x,y)=E_{n}(k_{z})\varphi_{n,k_{z}}(x,y).$$

where *H_kp_* is the k·p Hamiltonian matrix, *H_strain_* is the strain Hamiltonian matrix, *H_so_* is the split-off Hamiltonian matrix, which can describe spin-orbit coupling interaction, and *V*(*x*, *y*) is the channel potential. At the oxide interfaces, Dirichlet boundary conditions are applied to the wave functions, assuming that they do not penetrate the oxide.

**Figure 2.**
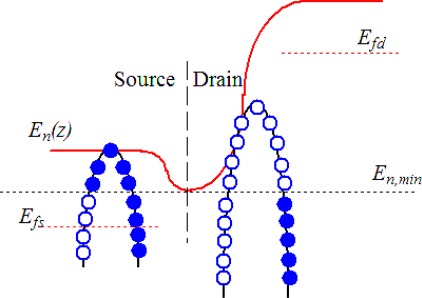
Illustration of the ‘top-of–the-barrier’ ballistic FET model. Semiclassical ballistic transport is assumed for the calculation of hole density and current. Filled circles represent states that are filled by the source Fermi level *E_fs_* and empty circles represent states filled by the drain Fermi level *E_fd_*.

Based on the effective valley degeneracy and the hole effective masses extracted from the bandstructures, which are calculated by using the 2-D discrete stress-dependent six-band k.p model, the ballistic I–V characteristics of the corresponding p-type SiNW FETs are evaluated by using a top-of-the-barrier ballistic FET model [23]. As shown in Figure 2, the subband of the p-type SiNW is coupled to the source and drain reservoirs, which are characterized by their Fermi levels.

In this way, this model treats ballistic transport semiclassically by filling the *k*-states at the top of the source-channel barrier and captures three-dimensional electrostatics, quantum capacitance, and bias-charge self-consistency in ballistic FETs. However, source-to-drain tunneling is not considered in this model. In this work, we assume the gate control parameter=0.88 and the drain control parameter=0.035, which indicates the gate control is not perfect, and can partly reflect the effect of the depletion capacitance due to the charge confinement at the center of the cross-section in the SiNWs [27]. Once the number of mobile carriers and the effective potential *U_scf_* at the top of the barrier are converged, the drive current is evaluated using the semiclassical transport equation in the ballistic limit:

(2) $$I=\frac{2q}{h}\int_{U_{\mathrm{scf}}}^{\infty }\mathrm{dE}[f(E_{\mathrm{fs}}-E(z))-f(E_{\mathrm{fd}}-E(z))].$$

where *f*(*E*) is the Fermi function.

## 3. Results and Discussion

**Figure 3.**
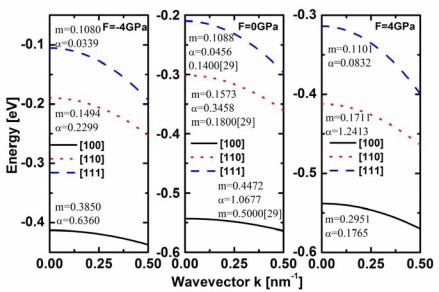
The energy dispersion relations for the first subband of the simulated strained and unstrained SiNWs with various channel orientations. T_Si_=W_Si_=2.2 nm.

For the valence band of the ultra-scaled cross section SiNW, the degeneracy between light and heavy holes is lifted by the strong quantum confinement. Because holes mainly occupy the first subband, Figure 3 only shows the first subband for the simulated strained and unstrained SiNWs with various channel orientations. It clearly shows that the bandstructures are different for SiNWs with various channel orientations, and the stress further modify the bandstructures, resulting in different hole effective masses.

To account for the influence of subband nonparabolicity on the effective mass, the following expression for zone-center effective mass *m*_//_ of SiNW is utilized [28]:

(3) $$\frac{\hbar^{2}k_{//}^{2}}{{2m}_{//}}=E_{//}(1+{\alpha E}_{//}+{2\alpha }^{2}E_{//}^{2})$$

where *E*_//_ is the hole energy measured from the subband edge, *k*_//_ is the hole wave vector, and α is nonparabolicity factor. Both the hole effective masses and nonparabolicity factors, also shown in Figure 3, are applied to investigate the orientation and the uniaxial stress dependence of the ballistic transport. Our calculated hole masses of unstrained SiNWs are slightly lower compared to the values calculated by using density function theory [29], due to the different simulation method, however, both methods present the similar result, i.e. when the width is 2.2 nm, the hole effective masses of the SiNWs along [110] and [111] directions reduce while that of the SiNW along [100] direction increases compared to the bulk silicon.

**Figure 4.**
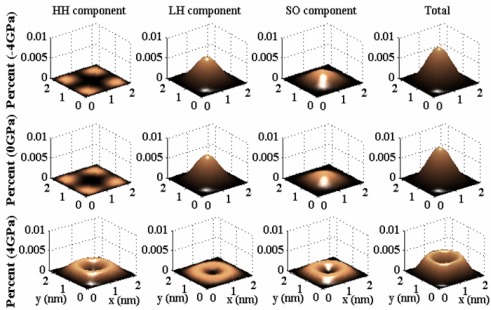
HH component, LH component, and SO component of the wave function of the ground state at *k_z_* = 0 in the simulated strained and unstrained SiNWs along [100] channel orientation.

In order to explain the origin of the phenomenon, in Figure 4, we first check the nature of the first subband of the SiNW along [100] orientation at the Γ (*k_z_* = 0) point. Under no stress, since the reduction of the dimensionality (stronger quantum confinement) enhances the band mixing effect [30], the amplitudes of the light hole (LH) and spin-orbit split-off (SO) wave functions have nonzero mixing, which results in pronounced nonparabolicity effect [14,31,32] (*α* = 1.0677*eV*^−1^). Because the more nonparabolicity effect of the same-oriented subbands is in the transport direction, the larger the transport mass will be [14], the first subband of the [100] SiNW has a heavier effective mass (0.4472) compared to the bulk silicon (*m*_*n*=1_ = 0.31). Under the stress, the wave function components are found to be sensitive to the tensile stress, which enhances the HH component and results in the band mixing effect among HH, LH, SO bands. However, this band mixing controlled by the tensile stress reduces nonparabolicity effect (*α* = 0.1765*eV*^−1^ due to quantum confinement, resulting in lighter effective mass (0.2951), in other word, the quantum confinement effect is partly nullified by the tensile stress. This can be seen from the change of the wave function shape, which reflects a different charge distribution in space. Under the tensile stress, the charge confined at the center of the cross-section relocalizes in the surface of SiNWs, which is similar as the classical case (*m*_*n*=1_ = 0.31).

**Figure 5.**
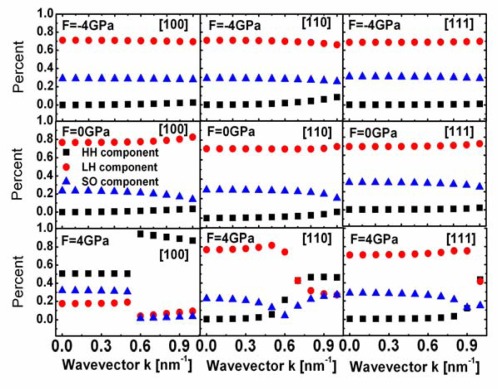
Percent of the HH, LH, SO components in the first subband of the simulated SiNWs versus wavevector *k*.

In Figure 5, we also check the percent of the HH, LH, SO components in the first subband of the simulated SiNWs with various channel orientations as a function of wavevector *k_z_*. It can be seen from Figure 5 that the first valence band of the SiNWs along [110] and [111] orientations is now determined by the light band at very large wavevector regions. Meanwhile, HH component is found to be zero. Because the LH bands of silicon along [110] and [111] orientations have very light effective hole mass [20] and nonparabolicity effect is small, the effective hole mass of the first subband of the SiNWs along [110] and [111] orientations is light, in spite of the mixing between LH and SO wave functions. Note that the variation of effective mass can also be seen from the change of shape/curvature of the valence bandstructure [14,20]. The larger dispersions’ curvature at energies close to the valence band edge, the lighter effective mass. The heaviest effective mass in the [100] SiNW, is a result of the enhanced warping in its dispersion, which reduces the dispersion’s curvature.

Changes in the bandstructure due to quantum mechanical confinement, strain and various transport orientations reflect on the transport characteristics of the strained SiNW with three wire orientations, [100], [110] and [111]. Based on the effective valley degeneracy and the effective masses extracted from the stress-dependent subbands, the top-of-the-barrier ballistic transport model is used to self-consistently calculate the drain current, carrier density and injection velocity of p-type SiNW transistors. Note that for the valence band of SiNWs along the [110] orientation, the band-edge of the second highest band is close to that of the highest band. So the second highest band also contributes to the hole density and current, and the effective valley degeneracy is close to 2. Besides, at the same doping concentration and temperature, the change of *E_V_* – *E_fs_* (due to different orientation and stress type wires) will result in a threshold voltage shift, where *E_V_* is the valence subband edge. However, to facilitate performance comparison, in this work, the threshold voltage (current value=1.45 μA/μm) of each device is calibrated to 0.25 V_DS_ by adjusting the gate work function. Thus, the threshold voltage shift will not be seen in the relative performances.

**Figure 6.**
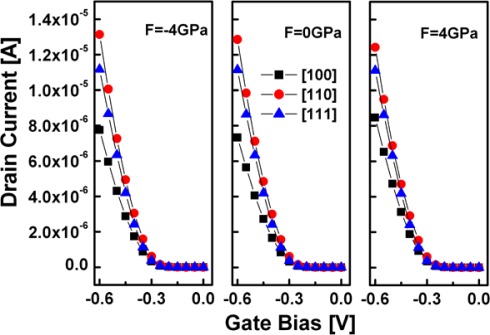
The I_DS_–V_GS_ curves for p-type strained and unstrained SiNW FETs with various channel orientations at V_DS_= – 0.6 V. The oxide thickness is assumed to be 1 nm and the temperature is 300 K.

Figure 6 plots the drive current vs. gate bias curves at V_DS_= – 0.6 V for 2.2 nm square SiNW FETs with three different channel orientations. The results show that the SiNW FET performance displays a strong orientation dependence. For p-type SiNW FETs, [110] is the optimum orientation that offers the highest drain current, followed by the [111] SiNW, whereas the [100] SiNW has the lowest drain current. On the other hand, the effect of strain on the ballistic drain current of SiNW FETs with the [110] and [111] orientations, except for the [100] orientation, turns out negligible in the case considered, which can be attributed to an only slight variation of the non-parabolic valence structure under the strong quantum confinement. In other word, in this case, the effect of the stress is nullified by quantum confinement.

**Figure 7.**
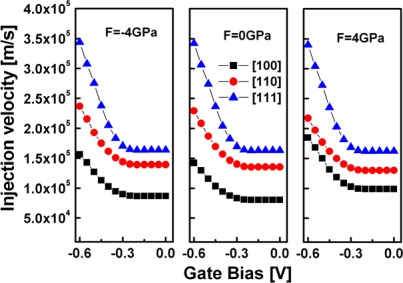
Injection velocity of the simulated strained and unstrained SiNW FETs with various channel orientations versus gate bias at V_DS_= – 0.6 V.

**Figure 8.**
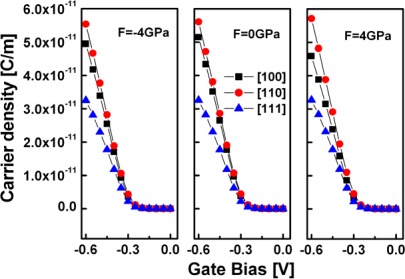
Carrier density of the simulated strained and unstrained SiNW FETs with various channel orientations versus gate bias at V_DS_= – 0.6 V.

Because current is the product of the carrier density and the injection velocity, in Figures 7 and 8, the injection velocity and the carrier density are plotted as a function of the gate bias, respectively. Figure 7 shows that the [111] SiNW has the largest injection velocities, followed by the [110] SiNW, whereas the [100] SiNW has the lowest injection velocities, as would be expected from the subband masses estimated earlier. However, from Figure 8, it is observed that the carrier density of high strained [111] oriented channel is obviously reduced, thus, degrades device performance, while the [110] SiNW has the largest carrier densities, thus, enhances device performance.

This result indicated the competition of injection velocity and carrier density related to hole effective masses plays a critical role in determining the performance of the nanotransistors. Furthermore, the effect of the stress on the injection velocity and the carrier density is also too small to be considered due to the strong quantum confinement.

**Figure 9.**
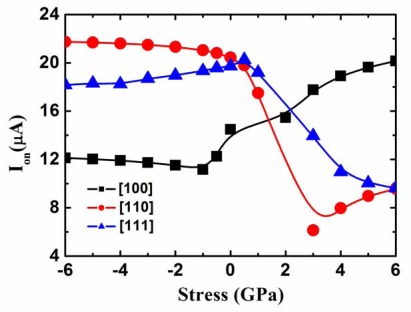
ON-current of the p-type 5 nm square SiNW FETs as a function of the stress.

As discussed above, due to the limit of the strong quantum confinement, the effect of the stress on the ballistic hole transport properties of the ultra-scaled 2.2 nm square SiNWs is almost too small to be considered. To gain further insight into the effect of the stress, we determine to investigate larger cross section SiNWs. Figure 9 shows ON-current (V_GS_=V_DS_= – 0.6 V) of the p-type 5 nm square SiNW FETs with a as a function of the stress. Because the degree of the competition between the stress and quantum confinement is different for different size wires, the 5 nm square SiNW for tensile stresses behaves in a different manner with respect to the 2.2 nm one.

In particular, although the uniaxial compressive stress slightly changes the ballistic hole current, the uniaxial tensile stress obviously alters the ballistic hole current in three different channel orientations. In other word, the effect of tensile stress is more obvious in the 5 nm one due to weaker quantum confinement effect. This clearly indicates that in order to obtain the high-sensitivity NW FET pressure sensors, the tensile stress should be applied.

Meanwhile, in order to enhance the effect of the stress, the size of the SiNW can not be too small or narrow, resulting in the strong quantum effect, which will counteract the stress effect. In addition, it can be seen from Figure 9 that [110] is the optimum orientation for the p-type SiNW FET pressure sensors, whereas [100] is the optimum orientation for the p-type SiNW FETs under the very high tensile stress level, which is attributed to the increase of the effective valley degeneracy of [100] SiNW.

**Figure 10.**
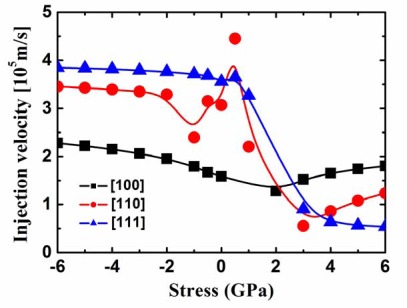
Injection velocity of the p-type 5 nm square SiNW FETs as a function of the stress.

Figure 10 plots the injection velocity vs. stress curves for the 5 nm square SiNW FETs with three different channel orientations. In opposition to the p-type 2.2 nm square SiNW FETs, the [100] SiNW has the largest injection velocities under the very high tensile stress, followed by the [110] SiNW, whereas the [111] SiNW has the lowest injection velocities.

**Figure 11.**
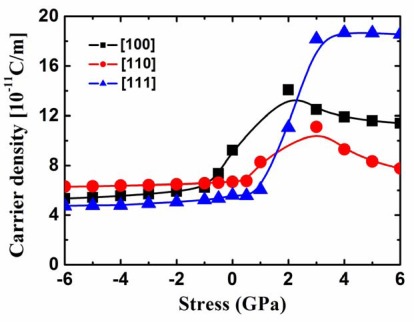
Carrier density of the p-type 5 nm square SiNW FETs as a function of the stress.

On the other hand, in Figure 11, it is observed that the high tensile-strained [111] oriented channel yields a very high carrier density, followed by the [100] oriented channel, whereas the [110] oriented channel has the lowest carrier density. This result is also different from that of the 2.2 nm square SiNW FETs. As discussed above, current is the product of the carrier density and the injection velocity, therefore, the hole transport property of the 5nm square SiNW is different from that of the 2.2 nm square SiNW under very high tensile stress level due to the different quantum confinement effects.

## 4. Conclusions

In order to design and optimize high-sensitivity SiNW FET sensors, we have provided a detailed examination of the impact of the orientations and the uniaxial stress on the hole subband structure and the relative ballistic transport characteristic of the p-type SiNW transistor by using the stress-dependent k.p method which is discretized with the nine-point finite difference method, and the top-ofthe-barrier ballistic transport model. It shows that SiNW FETs display a strong orientation dependence. We have identified [110] as the optimum orientation for the unstrained SiNW FETs. The dependence of SiNW FET performance on the uniaxial stress was also explored and the results show that there is a clear performance improvement when uniaxial tensile stress is applied along the transport orientation, and [110] is the optimum orientation for the p-type SiNW FET pressure sensors, whereas [100] is the optimum orientation for the very high tensile-strained p-type SiNW FETs. However, the effect of the uniaxial compressive stress on the ballistic drain current of SiNW FETs is small in the case considered, which can be attributed to an only slight variation of the non-parabolic valence structure under a compressive stress. Furthermore, it is observed that quantities related hole effective masses, such as carrier injection velocity and carrier density play key roles in determining the performance of p-type ballistic SiNW transistors.

## Figures and Tables

**Table 1.** Luttinger parameters, deformation potentials, spin-orbit split-off energy used in the calculation.

γ_1_	γ_2_	γ_3_	*a_v_* (eV)	*b* (eV)	*d* (eV)	Δ_so_ (meV)
4.22	0.39	1.44	2.46	−2.1	−4.8	44

## References

[1] Kim J.W., Takao H., Sawada K., Ishida M. (2007). Integrated inductors for RF transmitters in CMOS/MEMS smart microsensor systems. Sensors.

[2] Qiu Y.Y., Azeredo-Leme C., Alcacer L.R., Franca J.E. (2001). A COMS humidity sensor with on-chip calibration. Sens. Actuat. A.

[3] Lee S.P., Lee J.G., Chowdhury S. (2008). CMOS humidity Sensor system using carbon nitride film as sensing materials. Sensors.

[4] Hynes E., O’Neill M., McAuliffe D., Berney H., Lane W.A., Kelly G., Hill M. (1999). Development and characterization of a surface micromachined FET pressure sensor on a CMOS process. Sens. Actuat. A.

[5] Dai C.L., Tai Y.W., Kao P.H. (2007). Modeling and fabrication of micro FET pressure sensor with circuits. Sensors.

[6] Cui Y., Zhong Z.H., Wang D.L., Wang W.U., Lieber C.M. (2003). High performance silicon nanowire field effect transistors. Nano Lett.

[7] Cui Y., Wei Q.Q., Park H.K., Lieber C.M. (2001). Nanowire nanosensors for highly sensitive and selective detection of biological and chemical species. Science.

[8] Kobayashi M., Hiramoto T. (2008). Experimental study on quantum confinement effects in silicon nanowire metal-oxide-semiconductor field-effect transistors and single-electron transistors. J. Appl. Phys.

[9] Duan X., Niu C., Sahi V., Chen J., Parce J.W., Empedocles S., Goldman J.L. (2003). High-performance thin-film transistors using semiconductor nanowires and nanoribbons. Nature.

[10] Wang J., Rahman A., Ghosh A., Klimeck G., Lundstrom M.S. (2003). Performance evaluation of ballistic silicon nanowire transistors with atomic-basis dispersion relations. Appl. Phys. Lett.

[11] Ma D.D.D., Lee C.S., Au F.C.K., Tong S.Y., Lee S.T. (2003). Small-diameter silicon nanowire surfaces. Science.

[12] Wu Y., Cui Y., Huynh L., Barrelet C.J., Bell D.C., Lieber C.M. (2004). Controlled growth and structures of molecular-scale silicon nanowires. Nano Lett.

[13] Xiang J., Lu W., Hu Y., Wu Y., Yan H., Lieber C.M. (2006). Ge/Si nanowire heterostructures as high-performance field-effect transistors. Nature.

[14] Neophytou N., Paul A., Lundstrom M.S., Klimeck G. (2008). Bandstructure effects in silicon nanowire electron transport. IEEE Trans. Electron. Dev.

[15] Cho K.H., Yeo K.H., Yeoh Y. Y., Suk S.D., Li M., Lee J M., Kim M.K., Kim D.W., Park D., Hong B.H., Jung Y.C., Hwang S.W. (2008). Experimental evidence of ballistic transport in cylindrical gate-all-around twin silicon nanowire metal-oxide-semiconductor field-effect transistors. Appl. Phys. Lett.

[16] Sun G., Sun Y., Nishida T., Thompson S.E. (2007). Hole mobility in silicon inversion layers: stress and surface orientation. J. Appl. Phys.

[17] Luttinger M., Kohn W. (1955). Motion of electrons and holes in perturbed periodic fields. Phys. Rev..

[18] Chao C.Y., Chuang S.L. (1992). Spin-orbit-coupling effects on the valence band structure of strained semiconductor quantum wells. Phys. Rev. B.

[19] Fischetti M., Ren Z., Solomon P., Yang M., Rim K. (2003). Six-band k.p calculation of the hole mobility in silicon inversion layers: Dependence on surface orientation, strain and silicon thickness. J. Appl. Phys..

[20] Wang E.X., Matagne P., Shifren L., Obradovic B., Kotlyar R., Cea S., Stettler M., Giles M. D. (2006). Physics of hole transport in strained silicon MOSFET inversion layers. IEEE Trans. Electron. Dev.

[21] Zhang J.H., Huang Q.A., Yu H., Lei S.Y. (2008). Theoretical study of electromechanical property in a p-type silicon nanoplate for mechanical sensors. Chin. Phys. B.

[22] Seo W.H., Donegan J.F. (2003). 6×6 effective mass Hamiltonian for heterostructures grown on (11*N*)-oriented substrates. Phys. Rev. B.

[23] Rahman A., Guo J., Datta S., Lundstrom M. S. (2003). Theory of ballistic nanotransistors. IEEE Trans. Electron. Dev.

[24] Lassen B., Willatzen M., Melnik R., Lew Yan Voon L.C. (2006). Electronic structure of free-standing InP and InAs nanowires. J. Mater. Res.

[25] Maslov A.V., Ning C.Z. (2005). Radius-dependent polarization anisotropy in semiconductor nanowires. Phys. Rev. B.

[26] Park S.H., Ahn D., Lee Y.T. (2004). Finite element analysis of valence band structures in quantum wires. J. Appl. Phys.

[27] Marchi A., Gnani E., Reggiani S., Rudan M., Baccarani G. (2006). Investigating the performance limits of silicon-nanowire and carbon-nanotube FETs. Solid-State Electron.

[28] Foreman B.A. (1994). Analytic model for the valence-band structure of a strained quantum well. Phys. Rev. B.

[29] Vo T., Williamson A.J., Galli G. (2006). First principles simulations of the structural and electronic properties of silicon nanowires. Phys. Rev. B.

[30] Colak S., Eppenga R., Schuurmans M.F.H. (1987). Band mixing effects on quantum well gain. IEEE J. Quant. Electron.

[31] Rössner B., von Känel H., Chrastina D., Isella G., Batlogg B. (2007). Effective mass measurement: the influence of hole band nonparabolicity in SiGe/Ge quantum wells. Semicond. Sci. Technol.

[32] Gnani E., Reggiani S., Gnudi A., Parruccini P., Colle R., Rudan M., Baccarani G. (2007). Band-structure effects in ultrascaled silicon nanowires. IEEE Trans. Electron Dev.

